# Study Protocol of a feasibility and acceptability trial of Problem Management Plus with Emotional Processing (PM+EP) for forcibly displaced youth living in Sweden

**DOI:** 10.1136/bmjopen-2025-104845

**Published:** 2025-10-28

**Authors:** Erica Mattelin, Cansu Alozkan-Sever, Shervin Shahnavaz, Marit Sijbrandij, Ellenor Mittendorfer-Rutz, Aemal Akhtar

**Affiliations:** 1 Save the Children, Stockholm, Sweden; 2 Department of Public Health and Caring Sciences, Uppsala University, Uppsala, Sweden; 3 Amsterdam Public Health Research Institute, Amsterdam, Netherlands; 4 Department of Clinical, Neuro-, and Developmental Psychology, Vrije Universiteit Amsterdam, Amsterdam, Netherlands; 5 Department of Clinical Neuroscience, Centre for Psychiatry Research, Karolinska Institute, Stockholm, Sweden; 6 Department of Clinical Neuroscience, Division of Insurance Medicine, Karolinska Institutet, Stockholm, Sweden

**Keywords:** MENTAL HEALTH, Psychosocial Intervention, Refugees

## Abstract

**Introduction:**

Heightened rates of mental illness among children, young people and forcibly displaced adults are well-documented. Despite this, access to care in host countries is often low. Problem-management plus (PM+) is an intervention developed by the WHO that can be delivered through task-shifting by lay counsellors and has been shown to be effective in numerous studies. At the same time, it has been shown that PM+ has a limited effect on traumatic stress symptoms, a common problem among forcibly displaced individuals. In turn, to further these benefits, a novel emotional processing (EP) module has been developed to be adjunctively delivered alongside PM+(PM+EP).

**Method and analysis:**

The current study is a randomised controlled feasibility and acceptability study. 60 participants aged 16–25 will be randomly allocated to either PM+, PM+EP or care as usual. The primary outcome of this study will be the feasibility and acceptability of the delivery of PM+EP in forcibly displaced youth. Secondary outcomes are self-rated measures of distress, depression and anxiety, post-traumatic stress disorder, personally identified problems, hope, use of services and medications, general well-being and social support.

**Ethics and dissemination:**

Following ethical approval in February 2024, recruitment commenced in October 2024. Study completion is anticipated by December 2025. Findings will be disseminated via peer-reviewed publications, conference presentations and communication with relevant stakeholders.

**Trial registration number:**

NCT06878092.

**STRENGTHS AND LIMITATIONS OF THIS STUDY**

- The trial includes three intervention arms, enabling comparison of accessibility and feasibility between problem management plus (PM+), PM+ Emotional Processing and care as usual in Sweden.
- The aim of the study is to understand the feasibility of implementing scalable psychological interventions in the Swedish context, with the primary aim to understand the safety and acceptability.
- The study will be implemented through collaboration between academics and civil society to facilitate future translation from research to practice.
- Both researchers and assessors will be blinded to intervention allocation, minimising risk of bias.

## Introduction

 According to the UNHCR, there are currently more than 120 million people globally who have been forcibly displaced from their homes.^1^ More than 35% of those displaced have been forced to leave their home countries and have resettled elsewhere as refugees or asylum seekers.^1^ The most recent humanitarian crisis, taking place in Ukraine, has resulted in an additional 15.5 million people being forcibly displaced from their homes with more than 7 million leaving Ukraine; this has resulted in Europe having to manage the largest influx of non-refugee migrants, refugees and asylum seekers since the second World War.^2^ Sweden has historically had an inclusive migration policy, but following policy changes in 2015,^3^ asylum applications have declined. In 2024, 9645 individuals applied, excluding Ukrainians covered by the Temporary Protection Directive, the most common countries of origin being Syria, Afghanistan and Iraq.^3^ Individuals who have sought protection in Sweden from Ukraine are entitled to protection in Sweden if they left Ukraine no earlier than 22 February 2022 according to the Temporary Protections Directive. This is a residence permit that is currently valid until spring 2026.^4^

Alarmingly, 40% of those who have been forcibly displaced are children under the age of 18, many of whom are unaccompanied minors.^1^ Experiencing displacement has been shown to be associated with developing poor mental health outcomes.^5^ The prevalence of common mental disorders in both child and adult forcibly displaced populations has been shown to be higher than for host populations. In a recent meta-analysis, the prevalence of post-traumatic stress disorder (PTSD), depression and anxiety disorders among refugee children was 22.71%, 13.81% and 15.77%, respectively.^5^ Another study investigating mental ill health in young refugees resettled in high-income countries (HICs) found substantially higher numbers of refugees experiencing ill health compared with their non-refugee peers.^6^

There exists a troubling discrepancy between the increased occurrence of mental health issues among forcibly displaced youth and their access to mental health services. Research in HICs has demonstrated that forcibly displaced children and adolescents access mental health services less frequently than their counterparts in the host country.^7 8^ Barriers to seeking mental healthcare may include language problems, lack of knowledge about the health system, cultural differences and stigma.^9^ In Sweden, young migrants are less likely to receive a wide range of psychiatric diagnoses but also less likely to receive adequate treatment.^10^ Additionally, there are examples of existing laws in Europe that limit the ability of refugees and asylum seekers to access healthcare. In Sweden, adult asylum seekers older than 18 years have healthcare access limited to ‘treatment that cannot be deferred’, which excludes their rights to receive regular health care.^11^ There are several evidence-based psychological interventions that have been adapted for refugee populations, such as narrative exposure therapy,^12^ common elements treatment approach^13^ and multimodal interventions, such as family-based mental health promotion.^14^

Scalable psychological interventions that can be delivered by non-professional helpers have been developed and used as one option to address the large mental health treatment gap.^15^ Scalable interventions often use task-sharing approaches characterised by the redelegation of tasks performed by highly specialised mental health staff to those with less or no formal training, such as community members.^16^ Additionally, scalable interventions are often brief, transdiagnostic and focus on developing skills for self-management instead of treating disorders. The helpers who implement these interventions among forcibly displaced populations are often from similar ethnic, cultural, or language backgrounds.

One of the earliest developed scalable psychological interventions was problem management plus (PM+),^17^ which aimed to allow for mental health services to be delivered in places with limited mental health resources. PM+specifically targets people experiencing moderate levels of psychological distress and aims to relieve symptoms related to common mental disorders. The intervention is composed of evidence-based strategies related to problem-solving and behavioural activation and consists of four core components: (1) managing stress, (2) managing problems, (3) get going, keep doing, and (4) strengthening social support.^17^ Several studies have demonstrated the effectiveness of PM+ in adults residing in low-income and middle-income countries through individual ^18 19^ and group formats.^20^,^22^ More recently, the feasibility and effectiveness of the intervention in HICs among refugees have been explored in the Netherlands^23^ and Switzerland.^24^ Although no active ingredients targeting post-traumatic stress symptoms are included in the intervention, PM+ has been shown to reduce individual symptoms of PTSD.^25^ The emotional processing (EP) Theory proposes that effective psychosocial interventions to treat PTSD should include direct modification of the fear structure, which may give rise to symptoms of the disorder, by activating the fear structure using exposure and by integrating more realistic, non-threatening information into it.^26^ Further, recent evidence indicates that processing positive memories and imagining positive events in the future can positively affect mental health outcome.^27^ Given the pervasiveness of experienced traumatic events and PTSD in refugee populations, including in children and adolescents,^5^ it would be worthwhile exploring further whether exposure to memories of negative past events adds to the efficacy of a scalable intervention such as PM+ in reducing symptoms of PTSD and depression, and additionally as to whether it would be feasible to deliver active ingredients of exposure therapy using scalable psychological interventions. To further elucidate the contribution of the retrieval of negative memories in reducing PTSD symptoms, this study seeks to preliminarily compare PM+ with PM+ including an EP component (PM+EP).^28^ The primary aim of this feasibility randomised controlled trial (RCT) study is to evaluate the feasibility and acceptability of the PM+EP and PM+ interventions for forcibly displaced youth residing in Sweden. A secondary aim of this work is to preliminarily compare PM+ with an EP component with PM+ and care of usual.

## Methodology

### Study setting

The study will be implemented through Save the Children Sweden. Save the Children Sweden has 58 000 members organised in 130 local associations. The local associations are united across 25 districts. Local associations and districts work in their local areas to build activities for children, develop networks and have channels for local decision-makers. They are also involved in Save the Children’s various campaigns, advocacy work and international humanitarian work. Since 2015, Save the Children has also developed a system of case managers across the country working with children who require different kinds of support, often when in contact with the Swedish welfare system. The study will be situated in three of the four regions (South, East and West).

### Trial design

**Figure 1:**
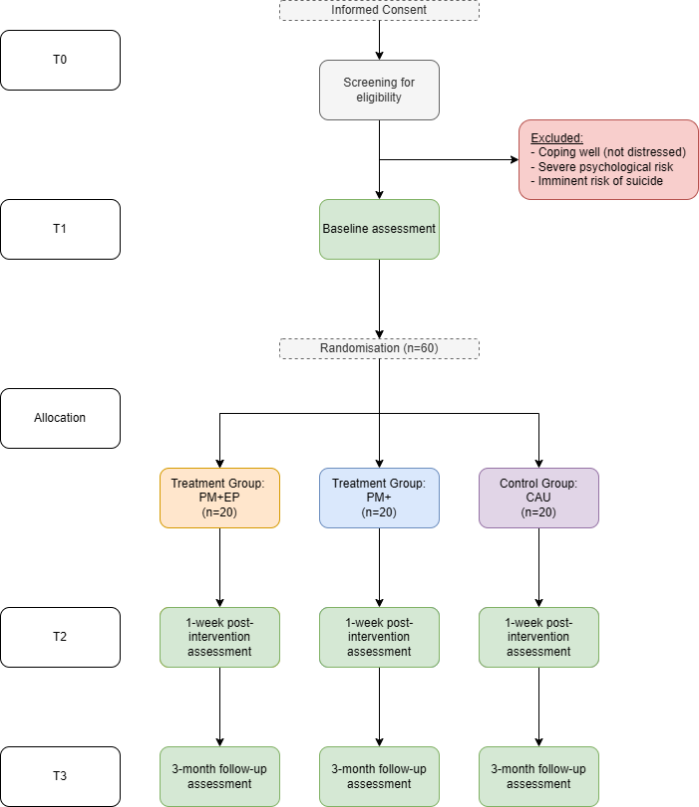
Study flow chart. CAU, care as usual; EP, emotional processing; PM, problem management.

The study consists of two phases: a feasibility RCT and a qualitative process evaluation. The feasibility RCT uses a three-armed single-blind randomised controlled parallel group design, comparing PM+, PM+EP and care as usual (CAU) for forcibly displaced persons living in Sweden. We aim to recruit 60 participants who screen positive for moderate psychological distress. Assessments will be conducted at baseline, 1 week after treatment completion (7 weeks) and 3 months after treatment completion (19 weeks). The flow chart of the trial is shown in figure 1. As part of the qualitative process evaluation, we will be exploring beneficiaries’ and implementers’ experiences with the intervention, and practitioners’ and policymakers’ thoughts on the use of PM+/PM+EP more widely in Sweden.

The study started in June 2024 and data collection is estimated to finish by December 2025.

### Inclusion criteria

Participants will be eligible for the trial if they meet the following criteria:

- Forcibly displaced youth aged 16–25 years old who arrived in Sweden after 2015.
- Elevated levels of psychological distress as determined through the Kessler-10 Psychological Distress Scale (K10; K10 >15).^29^
- Reduced levels of psychosocial functioning as measured by the WHO Disability Assessment Schedule (WHODAS 2.0>16).^30^
- Fluent in Swedish or in any language for which validated translations of the study manual and assessment tools were available.

### Exclusion criteria

Participants will be excluded from the study if they meet any of the following criteria:

- An acute medical condition.
- Imminent suicide risk (assessed by the PM+manual suicidal thought interview).
- Indication of psychotic disorders and substance dependence (assessed by PM+manual observation checklist).
- Indication of severe cognitive or neurological impairment (assessed by PM+manual observation checklist).
- In the case of current psychotropic medication used: change in dosage during the past 2 months.

Approximately 20 individuals will be recruited for the qualitative process evaluation research, with five individuals recruited from each of the following categories:

1. Forcibly displaced youth (16–25 years old) who received either the PM+EP or PM+intervention; both completers and dropouts will be included where feasible.
2. Family members or caregivers of trial participants.
3. PM+ and PM+EP facilitators/helpers who conducted the intervention.
4. Key informants, including but not limited to clinical supervisors of PM+/PM+EP facilitators, mental health specialists (eg,psychologists, psychiatrists), researchers and policy-makers.

### Informed consent

Potential participants in the study will be contacted by a member of the research team and will be provided with information about the study. Interested participants will be asked to provide informed consent prior to screening for potential participation in the trial. Participants will be asked to sign physical copies of the informed consent and to subsequently post the completed forms to Karolinska Institute (KI).

### Interventions

The trial will consist of three intervention arms: PM+, PM+EP and CAU.

### Problem management plus

PM+ is a brief, potentially scalable psychological intervention developed by the WHO and is delivered by non-specialised helpers.^17^ In brief, the intervention is based on cognitive behavioural therapy techniques and typically consists of 5 weekly, 90 min sessions. Participants are taught the following techniques during the sessions: stress management, problem-solving, behavioural activation and accessing social support (WHO, 2016). The intervention is described in detail elsewhere.^17^

For this study, a translated and adapted version (to the Swedish context) of PM+ will be used to match the exposure between the two active intervention arms. There will be 6 weekly 75 min sessions as opposed to 5. For the PM+arm, the additional session will include non-directive supportive counselling to match the time spent on the ‘emotional processing’ module.

### PM+ with EP module (PM+EP)

The PM+ strategies discussed above are identical across both interventions apart from the additional EP module.^28^ The additional module aims to provide a safe environment for participants to engage in the EP of both positive and negative memories. During the additional session, participants will be asked to imagine significant memories as pieces of a jigsaw puzzle; participants will be asked to elicit three memories, two positive/pleasurable and one negative/frightening. Participants will be assisted in processing these memories through discussing the puzzle pieces, the thoughts and emotions that come with the memory, and future perspectives. Participants will be asked to rate the intensity of emotions before and after they talk about their memories each time they are brought up.^28^ By constructing a life story, the participant is provided with the opportunity to adequately contextualise significant events, which may facilitate the recognition of interrelated emotional networks of experiences. Since this module will be added to a transdiagnostic intervention to reduce psychological distress, the distressing event may be a traumatic event, or any other adverse event (AE) with negative impact. Previous research has indicated that interventions involving exposure to traumatic memories can be safely administered to adolescents through the process of task-shifting.^31^ The additional module includes the following specific components:

1. Psychoeducation: Participants will be informed about the rationale behind the module and the mechanisms associated with it.
2. Choosing three pieces of their ‘jigsaw puzzle’ (memories): Participants will be asked to choose three memories, two of them being pleasurable memories and one difficult (distressing/frightening/upsetting) memory. Participants will be asked to give a label to each of these memories (eg, ‘leaving the country’, ‘meeting with best friend’).
3. EP of memories: After the ‘puzzle’ is ready, participants will be asked to talk about the memories on their lifelines. Participants will be asked to first discuss the positive memory and then the difficult memory. Talking about the same difficult memory will last for three sessions. Each time it is discussed, a discussion about a positive memory will follow.
4. Positive imagination for the future: The final part of this module aims to help participants to engage in positive thinking by asking them to imagine their future in a positive way.

The additional EP components will occur between sessions 3 and 6.

### Therapist competence and adherence

Both the PM+ and PM+EP interventions will be delivered by helpers with at least a high school degree, matched with the participants according to their language and cultural backgrounds. The helpers will be native speakers of the language that the participant speaks and will also have sufficient speaking ability in Swedish or English. Where participants prefer, native Swedish speaking helpers will be available to implement the intervention.

Prior to the study, helpers will receive a 9-day training that focuses on the PM+ and PM+EP strategies, with additional training on common mental health problems, basic counselling skills and self-care strategies. Following the training, helpers will be required to complete one practice case. Throughout their implementation of the intervention, helpers will receive clinical supervision (either individual or group supervision) weekly by a PM+/PM+EP trainer. Helpers will report adherence through two methods. First, they will fill in a self-rated fidelity checklist after each session. Second, audio recordings will be made for approximately 10% of the sessions, where participants consent to recording. The recordings will be scored independently by two research assistants.

### Care as usual

The comparison group will receive CAU. CAU for forcibly displaced youth (under the age of 18) in Sweden corresponds to primary healthcare, education, social protection services and specialised psychological treatment where required. However, adults are only able to access care that cannot be deferred. Once the individual has received their residence permit, they are able to access all the healthcare services offered. Save the Children has a large presence in providing social support and treatment for refugees nationally. This involves both psychosocial programming and having mental health clinics. For this study, participants will be recruited from ongoing psychosocial programming.

### Assessments

Table 1 presents all assessments and respective time points for administration. Screenings are conducted on paper and mailed to KI to be scored for inclusion. All other assessments will be administered remotely, with data entered directly through REDCap that will allow for the coding of instruments in multiple languages for ease of data collection and subsequent data analysis.

**Table 1:** Standard Protocol Items: Recommendations for Interventional Trials: schedule of enrolment, interventions and assessments for the PM+EP/PM+trial

	Measures	Enrolment (-T1)	Screening (T0)	Baseline (T1)	Intervention	Postassessment (T2; 7 weeks)	3-month follow-up (T3; 19 weeks)
Enrolment							
Informed Consent		X					
Eligibility screen			X				
Allocation				X			
Interventions							
PM+EP				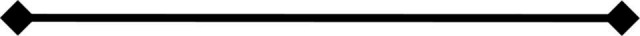			
PM+				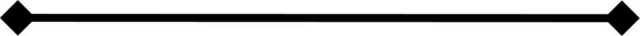			
CAU				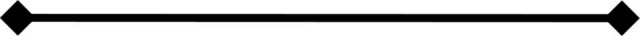			
Assessments							
Psychological distress	K10		X				
Impaired functioning	WHODAS 2.0		X				
Suicidal ideation	Suicide screener		X				
Depression symptoms	HSCL-25			X		X	X
Anxiety symptoms	HSCL-25			X		X	X
PTSD symptoms	PCL-5			X		X	X
Subjective well-being	WHO-5			X		X	X
Perceived problems	PSYCHLOPS			X		X	X
Agency	SHS-A			X		X	X
Social support	Bonding Social Capital			X		X	X
Post-migration stressors	PMLD			X		X	X
Traumatic experiences	TEC			X			X
Mental healthcare use	CSRI			X		X	X
Treatment fidelity	CSRI				X		

WHO PM+suicide screener (three items).

CSRI, Client Service Receipt Inventory; EP, emotional processing; HSCL-25, Hopkins Symptom Checklist (25-item); K10, Kessler psychological distress scale (10-item); PCL-5, PTSD Checklist for DSM-5; PM+, Problem Management Plus; PMLD, Post-Migration Living Difficulties; PSYCHLOPS, Psychological Outcomes Profiles; SHS-A, State Hope Scale (Agency Subscale); TEC, Traumatic Events Checklist; WHO-5, WHO-Five Well-Being Index; WHODAS 2.0, WHO Disability Assessment Schedule 2.0.

### Screening measures

The WHODAS 2.0 sociodemographic and disability assessment questionnaires will be collected at screening. The sociodemographic questionnaire will be used to collect information on age, sex, asylum status, marital status, education and work status, and living conditions. The WHODAS 2.0 is a questionnaire developed to measure health and disability.^30^ The 12-item version, which has been more commonly administered as a screening tool, will be used to understand levels of impaired functioning. Scores are rated on a 1 (none) to 5 (extreme) scale (range: 12–60), with higher scores indicating worsened levels of functioning. A score of 17 or higher will be used to indicate moderately impaired functioning. Minor adaptations of the questions have been made for this study to better suit youth populations.

The K10^29^ will be used to screen for levels of psychological distress. Items are scored on a scale of 1 (none of the time) to 5 (all of the time), with higher scores indicating higher levels of psychological distress experienced in the preceding month (range 10–50).^32 33^ To align with other PM+studies of refugees residing in HIC, we will use a cut-off score of 16 to indicate moderate levels of psychological distress.^23 24^

Finally, the imminent risk of suicide will be assessed using the three-item WHO PM+suicide screener.^34^ The first question posed to participants is whether ‘in the past month have (they) had serious thoughts or a plan to end your life?’. If the participant says no to this question, the questionnaire will end, and participants will be invited to participate in the trial dependent on their WHODAS 2.0 and K10 scores. If the participant says yes, two additional questions will be asked: (1) ‘Have you taken actions to end your life?’” and (2) ‘Do you plan to end your life in the next 2 weeks’. If the participant does not answer yes to these two questions, they will be included in the study and this information will be flagged to their PM+facilitator when providing support.

### Feasibility measures

The primary outcome of this study will be the feasibility and acceptability of the delivery of PM+EP in refugee youth. Feasibility will be determined through criteria related to consent and recruitment rates (>70%), attendance to PM+/PM+EP sessions (>70%), protocol adherence by helpers (>75%) and the number of AEs (<10%). In this study, AEs were defined as any unwanted experiences during the study, regardless of relation to PM+or PM+EP. Serious AEs (SAEs) are events causing death, life threat, hospitalisation or its prolongation, permanent disability or other significant medication conditions not necessarily connected to the intervention. All AEs/SAEs will be reported and followed, with specialist referral as needed. Regarding the feasibility of the research procedure, the completion of the baseline and follow-up assessments will be assessed (>85%). In addition, the qualitative process evaluation will further elucidate information of the acceptability of the interventions.

Protocol adherence will be determined through two methods. The first is a self-rated fidelity checklist that the PM+/PM+EP facilitators will fill out at the end of each session. This will contain each of the elements from the completed session, and facilitators will be asked to identify what items they completed and which items they may have missed to assess the degree of fidelity to the manualised intervention. second, audio recordings of sessions will be taken if participants provide informed consent for recording. A random sample of 10% of recordings will be scored independently by two research assistants.

### Secondary outcome measures

Secondary outcome measures will be administered at baseline and follow-up assessments to inform about possible effectiveness and preparation for a future definitive trial of PM+EP. The Hopkins Symptom Checklist-25 (HSCL-25) is a 25-item validated psychometric scale assessing symptoms of depression (15 items) and anxiety (10 items).^35^ Two of the depression items specifically relate to associated somatic symptoms. The items are rated on a 4-point Likert scale (range=25–100), ranging from 1 (not at all) to 4 (extremely).

PTSD symptom severity and diagnosis will be established with the PTSD Checklist for DSM-5 (PCL-5).^36^ The PCL-5 is a 20-item checklist, corresponding to the 20 symptoms of PTSD as described in the Diagnostic and Statistical Manual of Mental Disorders, Fifth Edition (DSM-5). Each item is scored on a 5-point scale, ranging from 0 (not at all) to 4 (extremely). When the symptom severity score is rated as two (moderate/threshold) or higher, a symptom is considered present. Total symptom severity is calculated by summing the scores of the 20 items (range: 0–80), with higher scores indicating worsened severity of post-traumatic stress symptoms.

The Psychological Outcomes Profiles (PSYCHLOPS) will be used to indicate the client’s perspective of change in their psychological distress level after therapy.^37^ PSYCHLOPS has four questions measuring three domains; the problem(s) bothering the individual (two questions), and how these problems affect the daily functioning and well-being. Scores range from 0 to 5 and higher scores represent more psychological difficulty (range=0–20).

Social support will be measured through a 1-item question assessing ‘bonding social capital’ from the Stockholm Public Health Cohort.^38^ Participants will be asked: ‘Do you know any people who can provide you with personal support for personal problems or crises in your life?’. The response will be categorised as either high social support or low social support.

The Client Service Receipt Inventory (CSRI)^39^ will be used to track the healthcare service use and medications that the participant has received in the 3 months preceding their entry to the study and over the course of the study.

The WHO 5-item well-being Index (WHO-5) will be used to measure subjective general well-being. The questionnaire is a 5-item scale assessing general psychological well-being and quality of life as opposed to well-being tied to a certain psychopathology.^40^ Items are scored on a 6-point scale ranging from 0 (at no time) to 5 (all of the time); total scores are the sum of the individual items ranging from 0 to 25, with higher scores corresponding to increased well-being.

Lastly, the Agency-Subscale State Hope Scale (Adult version; SHS-A) will be used to measure hope among study participants.^41^ The agency subscale of the SHS is a three-item scale measuring goal-directed behaviour during the intervention. Items are scored on an 8-point Likert scale ranging from 1 (definitely false) to 8 (definitely true). Total scores for agency are calculated by summing the item-level scores (range: 3–24), with higher scores indicating more goal-directed behaviours.

### Other measures

Exposure to traumatic events will be assessed through a Traumatic Events Checklist. The checklist contains 29 forms of traumatic events that the participants may have experienced during their displacement and subsequent travel to Sweden. The checklist was developed and used among refugee populations residing in several contexts^20 23 24 42^ and further adapted to the Swedish context prior to use.

The Post-Migration Living Difficulties Checklist (PMLD)^43^ will be used to measure the level of postmigration challenges experienced by the participant during the last 12 months. The checklist contains 17 items measured on a 5-point scale ranging from 0 (not a problem) to 4 (a very serious problem). Items that are either scored 3 or 4 are considered positive responses indicating a living difficulty. A total count of living difficulties is computed by summing the number of items, scoring either 3 or 4. For this study, two new items will be added to the list: (1) being bullied or rejected by peers and (2) arguments with parents over new friends and/or hobbies. Additional adaptations include adapting the item difficulties with employment, difficulties with education/employment and conflicts with social workers/other authorities to include teachers.

### Assessor competence

Data will be collected remotely through telephone assessments. The assessors undergo a 2-day training on questionnaire administration, basic interviewing skills, common mental health disorders, psychological first aid and ethics of research. In addition to this, assessors will have the opportunity to receive supervision on needs for referral from clinical staff and on the assessments by the trainer. Assessors will be available for all languages included in the study. All assessors should be fluent in either Swedish or English. They will be blinded to the allocation of the participants; during follow-up assessments and subsequent to the intervention period, assessors will instruct participants not to reveal the intervention that they received. The assessors will be recruited by Save the Children Sweden.

### Study procedure

Potential participants will be recruited through ongoing Save the Children Sweden activities in three regions of Sweden (East, South and West). Save the Children Sweden has available programmes for children, young people and caregivers nationally. In 2024, activities reached almost 40 000 children and young people. These programmes included psychosocial activities, group-based mental health support and safe meeting places where displaced persons were able to receive support. Recruiting staff at Save the Children will inform all young people about who may meet the eligibility criteria about the possibility of participating during their regular activities. If recruitment for the final sample size is not possible through ongoing Save the Children services, advertisements will be placed on social media with information about the study. Potential participants who indicate interest in participation are then referred to the study team and perform an initial screening assessment of eligibility using the WHODAS 2.0, K10 and a structured suicide risk evaluation assessment. Participants will be invited to the baseline assessment if they meet the eligibility criteria stated above and consent to participating in the trial. At baseline, participants will complete the following assessments: HSCL-25, PCL-5, PSYCHLOPS, WHO-5, bonding social capital, SHS-A and CSRI. In addition, participants will be asked to complete PMLD and the traumatic event checklists. Following the assessment, participants will be randomly assigned in a consecutive manner, using a 1:1:1 ratio for the randomisation process. The randomisation sequence will be computed by an independent researcher from KI using computerised software and allocation will be conducted by Save the Children Sweden; allocation is concealed from all study staff. Following the completion of the intervention, participants will be invited to complete 1-week postintervention (7-week) and 3-month follow-up (18-week) assessments.

After the 3-month follow-up, participants will be invited to take part in the qualitative process evaluation. Individual semistructured qualitative interviews will be conducted by researchers and psychologists. Interviews with participants and their families will be held in their preferred language. For key informants, interviews will be conducted on an ongoing basis throughout the trial, and for PM+EP/PM+helpers, at the conclusion of their final sessions.

On the completion of the trial, participants identified as requiring more support will receive information and assistance in locating suitable and appropriate follow-up care. For those who were randomised into CAU, PM+ will be offered after the 3 months post-assessment.

### Sample size

Since this is a pilot feasibility trial, no formal power calculations have been carried out. A total number of 60 forcibly displaced youth (20 in each arm) will be included in the study, allowing us to evaluate the feasibility and acceptability of the intervention in the proposed setting, as well as drop-out rates for a future definitive trial. This sample size was determined to be sufficient based on prior similar studies.^44 45^

### Analysis plan

The primary analyses for the feasibility RCT are planned to understand the safety and acceptability of the intervention and to preliminarily understand the within-group changes prior to and following the PM+(EP) Interventions. To accomplish this, both quantitative and qualitative methods will be used. The data will be analysed for the primary outcome and the other continuous measures using repeated measures analysis of variance. Kruskal-Wallis tests will be used if the normal distribution assumption fails. For the feasibility evaluation, intervention-specific analysis (fidelity, attendance to sessions) will be carried out. The qualitative process evaluation data will be analysed following thematic analysis through NVivo Scientific Software. To estimate potential effectiveness for a future trial, intention-to-treat analyses will be employed. Cohen’s d, based on pooled SD, will be used to calculate the treatment effect size of the PM+EP (based on primary outcome).

### Data management and confidentiality

Participants will complete questionnaires through the online platform REDCap hosted on KI’s secure computing facilities, with data backup handled by the same institute. The platform ensures additional security through two-factor authentication. The digital data, including audio files, are stored on encrypted servers, requiring a VPN connection, a personal service card and/or two-step authentication in accordance with local data management guidelines. The analogue data gathered during the study are securely stored within a locked file cabinet. Access to this cabinet is limited to the principal investigator, who holds the key. Additionally, entry to the archive room corridor necessitates a personal service card, and access to the room itself requires a passcode. This ensures the protection of the data’s confidentiality and integrity.

The data collected in the study will be handled and stored in accordance with the Research Data Management Policy of KI in compliance with both Swedish and European legislation. All study personnel involved in the trial will also have signed a confidentiality agreement.

### Patient and public involvement

During the conceptualisation of the research procedure, members of the relevant communities of interest were recruited through Swedish civil society to receive feedback on how recruitment could be conducted to ensure that those who would benefit the most from participation would be identified. Furthermore, community feedback was sought to ensure that there was a need for task-sharing interventions in migrant and refugee communities, and that the implementation would be positively viewed. Finally, Swedish psychologists with extensive experience working among and providing direct services in these communities will be involved throughout this work to provide feedback on the interventions of interest and to ensure that the materials were being appropriately culturally adapted prior to use.

### Ethics and dissemination

The trial was approved by the Swedish Ethical Authority (ID: 2023-07827-01). The trial was registered at Clinicaltrials.gov; NCT06878092 on 14 March 2025. All participants will provide informed consent for participation in the trial phase prior to completing the screening process. Data will be reported in accordance with the Consolidated Standard of Reporting Trials statement for non-pharmacological trials (CONSORT). The outcomes of this study will be disseminated through peer-reviewed scientific presentations at academic conferences, and targeted communication to non-governmental organisations, policy-makers and the broader public in Sweden and internationally.
