# Supplementary material for: Study Protocol of a feasibility and acceptability trial of Problem Management Plus with Emotional Processing (PM+EP) for forcibly displaced youth living in Sweden
Source: BMJ Open. 2025 Oct 28;15(10):e104845. doi: 10.1136/bmjopen-2025-104845 (PMC12570895; doi:10.1136/bmjopen-2025-104845)
Supplement: Supplementary file 1 [file bmjopen-2025-104845.reviewer_comments.pdf]

PEER REVIEW HISTORY

BMJ Open publishes all reviews undertaken for accepted manuscripts. Reviewers are asked to complete a checklist review form and are provided with free text boxes to elaborate on their assessment. These free text comments are reproduced below.

ARTICLE DETAILS

Title (Provisional)

Study protocol of a feasibility and acceptability trial of Problem Management Plus with Emotional Processing (PM+EP) for forcibly displaced youth living in Sweden

Authors

Mattelin, Erica; Alozkan-Sever, Cansu; Shahnavaz, Shervin; Sijbrandij, Marit; Mittendorfer-Rutz, Ellenor; Akhtar, Aemal

---

VERSION 1 - REVIEW

**Reviewer**	**1**
**Name**	**Benoni, Roberto**
**Affiliation**	**University of Rome La Sapienza**
**Date**	**06-Aug-2025**
**COI**	**None**

This article discusses the feasibility and acceptability of an intervention based on PM+ proposed by the WHO with the addition of an emotional education component to improve the mental health of the migrant population. The protocol is interesting and deals with a current and important issue, but some revisions are necessary:

- The authors should rewrite the objectives more clearly. It is unclear whether the primary objective is to independently verify the acceptability and feasibility of the PM+ and PM+EP interventions or to compare them. Furthermore, they should specify in the objectives whether they also intend to compare their effectiveness with each other and with standard care.

- In the introduction, it might be useful to highlight other interventions carried out on adolescents and young adults to improve mental health through social and emotional learning (see also DOI: 10.7189/jogh.14.04182)

- The authors report the significant population movement from Ukraine following the Russian-Ukrainian conflict, but provide general data on PTSD. It may be useful to provide contextualised data on this specific population (see also DOI: 10.1186/s12955-024-02226-1)

- In the study setting, it might be useful to provide more details on the number of migrants present in Sweden and on the specific mental health services available.

- In addition to the two strategies (PM+ and PM+EP), the authors should briefly clarify what 'care as usual' means in the Swedish context.

- The inclusion/exclusion criteria do not clearly state whether there will be restrictions related to country of origin. This is an important point, particularly in order to verify whether the questionnaires that will be used for the various assessments are available and validated in the native language of the people included.

- It should be specified more clearly how acceptability will be assessed. Will it be assessed solely using qualitative methods, or will quantitative criteria be used as for feasibility?

- The authors state that feasibility will be assessed as adverse events <10%, but they do not explain how 'adverse events' will be defined in this specific context.

- It is unclear how potential participants will be recruited. Will all individuals involved in Save the Children activities in the three regions of Sweden be asked to participate in the study? If not, who will select the individuals to be asked to participate, and how? Approximately how many individuals participate in these activities and therefore form the population from which the sample size will be drawn? Do they have any particular characteristics that could affect the results?

- The specific methodology for the qualitative part is missing. Will focus groups or interviews be conducted? Who will conduct them? In either case, it would be useful to provide an outline of the questions that will be used.

It would be advisable for the authors to discuss the possible limitations of the study and any measures taken to try to limit bias.

Hope this help.

VERSION 1 - AUTHOR RESPONSE

Responses to Reviewer: 1

Dr. Roberto Benoni, University of Rome La Sapienza

Comments to the Author:

This article discusses the feasibility and acceptability of an intervention based on PM+ proposed by the WHO with the addition of an emotional education component to improve the mental health of the migrant population. The protocol is interesting and deals with a current and important issue, but some revisions are necessary:

1. Comment: The authors should rewrite the objectives more clearly. It is unclear whether the primary objective is to independently verify the acceptability and feasibility of the PM+ and PM+EP interventions or to compare them. Furthermore, they should specify in the objectives whether they also intend to compare their effectiveness with each other and with standard care.

Response: Thank you for this comment. To ensure that the objectives of the feasibility trial are clearer, we have adjusted to language at page 5, lines 122-128 to ensure that readers know that the primary objective is to test the feasibility and acceptability of the interventions, individually and that the secondary objective is to preliminary compare the three arms of the trial.

2. Comment: In the introduction, it might be useful to highlight other interventions carried out on adolescents and young adults to improve mental health through social and emotional learning (see also DOI: 10.7189/jogh.14.04182)

Response: Thank you for this suggestion, as we agree that it is important to highlight other interventions that have been tried in younger adults. These changes can be found on Page 4, line 87-91. The majority of interventions endorsed by WHO have guidelines indicating interventions developed for adult (including younger adult) populations are appropriate to use at ages 16 or higher. In turn, in our update we focused on including further information on interventions that are available for use in this population. For younger populations, including children and early adolescents, different interventions are recommended (e.g. EASE in early adolescents, certain formats of CETA, etc). These were not specified in the updates as they are not directly relevant to the populations recruited as part of this work.

3. Comment: The authors report the significant population movement from Ukraine following the Russian-Ukrainian conflict, but provide general data on PTSD. It may be useful to provide contextualised data on this specific population (see also DOI: 10.1186/s12955-024-02226-1)

Response: Thank you for this comment. Considering the editor's feedback—which we agree was valid—we have aimed to maintain a broader scope rather than focusing solely on the Ukrainian population. However, if the editor would prefer that we include Ukraine-specific data, we are happy to make that update.

4. Comment: In the study setting, it might be useful to provide more details on the number of migrants present in Sweden and on the specific mental health services available.

Response: Thank you and we agree on this. We have included information about the number of migrants in the background on page 3, line 62-68 and information about the specific mental health services available in the care as usual section (see comments below) on page 9, lines 247-255.

5. Comment: In addition to the two strategies (PM+ and PM+EP), the authors should briefly clarify what 'care as usual' means in the Swedish context.

Response: We have now included a section on this on page 9, line 247-255 to further provide information on the available services for migrant and refugee populations in Sweden.

6. Comment: The inclusion/exclusion criteria do not clearly state whether there will be restrictions related to country of origin. This is an important point, particularly in order to verify whether the questionnaires that will be used for the various assessments are available and validated in the native language of the people included.

Response: We thank the reviewer for this comment and have attempted to ensure the text is more clear in the manuscript. There were no restrictions to country of origin, which meant that the intervention manual and all questionnaires had to be available in their language. Given the Swedish cntext, this meant that the PM+/PM+EP manuals, questionnaires, and all information provided through CAU had to be available in Ukrainian, Arabic, Dari, English, and Swedish.

7. Comment: It should be specified more clearly how acceptability will be assessed. Will it be assessed solely using qualitative methods, or will quantitative criteria be used as for feasibility?

Response: Acceptability will only be assessed with qualitative methods. We have tried to make this more clear at page 10, starting on line 287.

8. Comment: The authors state that feasibility will be assessed as adverse events <10%, but they do not explain how ‘adverse events’ will be defined in this specific context.

Response: Thank you for spotting this. We have added further information on how (serious) adverse events were defined in this work on page 10, lines 291-296.

9. Comment: It is unclear how potential participants will be recruited. Will all individuals involved in Save the Children activities in the three regions of Sweden be asked to participate in the study? If not, who will select the individuals to be asked to participate, and how? Approximately how many individuals participate in these activities and therefore form the population from which the sample size will be drawn? Do they have any particular characteristics that could affect the results?

Response: We thank the reviewer for this important comment. We have provided further information on the types of activities and national reach of Save the Children activities on Page 13, line 370-376. The authors do not believe that these particular characteristics will affect the results. The trial is meant to be pragmatic in the sense that the interventions are being embedded in ongoing programming, with the primary aims being safety and acceptability with qualitative work

10. Comment: The specific methodology for the qualitative part is missing. Will focus groups or interviews be conducted? Who will conduct them? In either case, it would be useful to provide an outline of the questions that will be used.

Response: Thank you for this comment. We have tried to expand on this on page 14, lines 392-396.

11. Comment: It would be advisable for the authors to discuss the possible limitations of the study and any measures taken to try to limit bias.

Response: Thank you for this input. As per the editors' suggestions, we have now removed the discussion section to adhere to the journal requirements.
